# Relation Between Anaerobic Power and Competitive Performance in Paralympic Powerlifting Athletes: A Cross-Sectional Study

**DOI:** 10.3390/jfmk11030263

**Published:** 2026-07-01

**Authors:** Carolina Oliveira de Carvalho, Felipe J. Aidar, Vanessa Carla Monteiro Pinto, Paulo Moreira Silva Dantas, Gilmara Gomes de Assis, Jonathas Helber Souza Santos, João Pedro de Macêdo Barros, Júlio César Medeiros Alves, Paulo Francisco de Almeida-Neto, Luis Leitão, Breno Guilherme de Araújo Tinoco Cabral

**Affiliations:** 1 Department of Physical Education, Federal University of Rio Grande do Norte, Natal 59078-900, Rio Grande do Norte, Brazil; 2 Department of Physical Education, Federal University of Sergipe (UFS), São Cristóvão 49100-000, Sergipe, Brazil; fjaidar@gmail.com; 3 Health Sciences Center, Federal University of Rio Grande do Norte, Natal 59012-570, Rio Grande do Norte, Brazil; vanecmpinto@gmail.com (V.C.M.P.);; 4 Escola Superior Desporto e Lazer, Instituto Politécnico de Viana do Castelo, Rua Escola Industrial e Comercial de Nun’Álvares, 4900-347 Viana do Castelo, Portugal; 5 Instituto Politécnico de Setúbal, Escola Superior de Educação, CIEQV, 2910-761 Setúbal, Portugal; 6 Life Quality Research Centre (CIEQV), 2910-761 Setúbal, Portugal; 7 Research Centre in Sports, Health and Human Development (CIDESD), 6201-001 Covilhã, Portugal

**Keywords:** paralympic powerlifting, anaerobic threshold, wingate test, athletic performance

## Abstract

**Background**: Paralympic powerlifting requires high upper-limb force production within a short time frame, making anaerobic power a potentially relevant physiological component associated with competitive performance. This study aimed to analyze the associations between biodynamic variables and competitive performance in Paralympic powerlifting athletes. **Methods**: A cross-sectional study was conducted with 13 elite Paralympic powerlifters. Body composition was assessed using DXA. Arm isometric strength was measured by the handgrip test, dynamic strength by the medicine ball throw, and anaerobic power by an upper-limb-adapted Wingate test. Competitive performance was determined based on the official ranking position in the Brazilian Paralympic Powerlifting Championship. **Results**: Significant correlations were observed between all anaerobic power variables and competitive performance: relative mean power (r = −0.864; *p* < 0.001), mean power (r = −0.804; *p* = 0.003), relative peak power (r = −0.766; *p* = 0.006), and peak power (r = −0.791; *p* = 0.004). Additionally, total lean mass and left-hand grip strength showed significant associations with maximum competition lift in exploratory linear regression analyses. **Conclusions**: Upper-limb anaerobic power showed strong associations with competitive performance indicators in Paralympic powerlifting athletes. Furthermore, lean mass and upper-limb isometric strength showed significant associations with maximum competition lift, supporting the multifactorial nature of competitive performance in Paralympic powerlifting. These findings suggest the potential relevance of integrating power-, strength-, and body composition-oriented assessments and interventions into the training process of elite Paralympic powerlifters.

## 1. Introduction

Paralympic Powerlifting (PP) is one of the fastest-growing Paralympic sports and is characterized by high demands of upper-limb strength and performance. Competitive success depends on the ability to lift the maximum load in the bench press according to official competition rules and body-weight categories [1]. Due to the structure of the sport and classification system, athletes compete within weight classes, which may influence performance interpretation and ranking outcomes.

Paralympic Powerlifting is organized into body-weight categories, in which athletes compete within specific classes according to official regulations. Therefore, competitive performance outcomes may be influenced not only by physiological characteristics but also by weight-category context [2]. Considering that competitive performance in the present study was operationalized as ranking position, the potential influence of body-weight categories should be considered when interpreting the findings.

Paralympic athletes present heterogeneous impairment profiles that may influence physiological and performance responses. Factors such as impairment type, body asymmetries, functional limitations, and altered movement patterns may affect force production, body composition characteristics, and competitive performance [3]. Therefore, the relationship between physiological variables and competitive performance in Paralympic athletes may differ from that observed in able-bodied populations, reinforcing the need for population-specific investigations.

Upper-limb anaerobic power has been considered an important physiological component in sports involving short-duration and high-intensity efforts. In Paralympic Powerlifting, the ability to produce and sustain power may contribute to competitive performance, particularly considering the upper-limb demands of the modality [3]. Therefore, assessments such as the Wingate test may provide useful information regarding athletes’ anaerobic profile and performance-related characteristics.

Traditionally, maximal strength has been considered an important component of success in powerlifting disciplines. However, evidence suggests that performance in Paralympic contexts is multifactorial, involving not only strength but also body composition characteristics and physiological factors related to performance [4,5]. In this context, assessments of anaerobic power and body composition may contribute to understanding competitive performance in Paralympic Powerlifting athletes. These aspects are especially relevant in Paralympic athletes, whose heterogeneous impairment profiles may influence body composition, movement patterns, functional capacity, and force production characteristics. Differences related to impairment type, asymmetries, and functional limitations may contribute to variability in physiological responses and competitive performance. Consequently, the relative contribution of performance determinants may differ from that observed in able-bodied athletes, reinforcing the need for population-specific investigation.

The Wingate test adapted for the upper limbs has been widely used to assess anaerobic power and has demonstrated validity and reliability in populations with physical disabilities [6,7,8,9,10,11]. This test provides important indicators such as peak power, mean power, and fatigue index, which can help characterize the anaerobic profile of athletes [12,13,14,15,16,17,18]. Moreover, its capacity to simulate short-duration, high-intensity efforts enhances its relevance for sports requiring rapid force production [19,20,21,22,23,24]. Despite its applicability, there is still a scarcity of studies investigating how these variables relate specifically to performance in Paralympic powerlifting competitions. Moreover, most existing studies have focused on able-bodied populations, limiting the generalizability of findings to Paralympic athletes and underscoring the need for context-specific evidence. In addition, available evidence rarely considers important contextual factors, such as body-weight categories and impairment heterogeneity, which may influence performance interpretation and the relationships between physiological variables and competitive outcomes.

Furthermore, the relationship between biodynamic variables—including body composition, assessed by dual-energy X-ray absorptiometry (DXA), handgrip strength, medicine ball throw performance, and upper-limb anaerobic power assessed through the Wingate test—and competitive outcomes remains insufficiently explored in Paralympic Powerlifting athletes. A better understanding of these associations may contribute to the interpretation of physiological and performance-related characteristics in this population and support evidence-based approaches for performance monitoring.

Importantly, understanding the associations between biodynamic variables and competitive outcomes may contribute to improving performance interpretation and monitoring in Paralympic Powerlifting [25,26,27,28]. Considering that competitive performance in the present study was operationalized as ranking position, investigating physiological variables associated with performance may provide relevant information regarding athlete characteristics and performance-related profiles in this population. Such an approach is especially pertinent in Paralympic populations, where heterogeneity in impairment type and functional capacity may influence the interaction between physiological variables and competitive performance. However, although relevant, impairment heterogeneity was not controlled in the present study and should therefore be considered when interpreting the findings.

Therefore, the aim of this study was to analyze the associations between biodynamic variables related to body composition, strength, and anaerobic power and competitive performance, operationalized as ranking position, in Paralympic powerlifting athletes. Additionally, exploratory secondary analyses were conducted using maximum competition lift as a complementary performance outcome.

## 2. Materials and Methods

### 2.1. Study Design and Participants

This cross-sectional study included a sample of 13 Paralympic Powerlifting athletes of both sexes competing at national and international levels. According to the International Classification of Athletes [29], 15.4% were classified as Level 5 (Paralympic), 15.4% as Level 4 (International), and 69.2% as Level 3 (National). Participants were eligible according to official Paralympic Powerlifting regulations and presented heterogeneous impairment profiles. The impairments represented in the sample included dwarfism (2 athletes), unilateral and bilateral lower-limb amputations with different levels of involvement, poliomyelitis affecting the lower limbs, tibial agenesis, spinal cord injury due to firearm injury (SCI) at T4 and T6–T8 levels, paraplegia due to firearm injury at T9–T10 levels, and arthrogryposis.

Participants were recruited from Paralympic Powerlifting training centers and were actively engaged in the modality during the data collection period. Inclusion criteria were: (a) active participation in Paralympic Powerlifting competitions; (b) previous experience in the modality; (c) regular training practice during the data collection period, with a minimum frequency of three weekly sessions lasting approximately two hours each; and (d) eligibility according to official Paralympic Powerlifting regulations. Exclusion criteria were: (i) presence of acute injuries or medical conditions preventing test completion; and (ii) failure to attend all evaluation stages.

To obtain results representative of pre-competition conditions and to avoid confounding effects related to overload or inadequate recovery, assessments were conducted 15 days prior to the 2024 Brazilian Powerlifting Championship, officially sanctioned by the Brazilian Paralympic Committee (5–6 December 2024) [13]. During this period, athletes were actively engaged in regular training routines and were in the pre-competitive phase; however, tapering strategies, recent training load, and recovery status were not formally monitored. Therefore, potential variations related to fatigue and pre-competition adjustments should be considered when interpreting the findings. Participants received consistent verbal encouragement during all testing procedures.

Participants were instructed to maintain their usual training, sleep, and nutritional routines during the assessment period, as evaluations were conducted in the pre-competition phase. No specific restrictions regarding caffeine intake, hydration status, nutritional intake, or upper-limb training were imposed before testing. Therefore, the potential influence of these factors on anaerobic performance outcomes should be considered when interpreting the findings.

The sample size calculation was performed a priori based on results from a previous unpublished pilot study (*n* = 10) using the open-source software G*Power (Version 3.0; Berlin, Germany) [24]. An α level of 0.05 and a statistical power of β = 0.90 were adopted. The estimated effect size (R^2^ = 0.512) was based on the two-tailed correlation between mean power measured in the upper-limb Wingate test and athletes’ ranking in a national competition. The analysis indicated a statistical power of 0.91. Due to the limited sample size and the exploratory correlational design of the study, analyses were not stratified according to sex or impairment profile. Therefore, findings should be interpreted considering the potential influence of these variables on physiological responses and competitive performance.

The study complied with all relevant national regulations and institutional policies, followed the principles of the Declaration of Helsinki, and was approved by the local Ethics Committee of the Federal University of Rio Grande do Norte (approval number: #83312924.5.0000.5537).

### 2.2. Procedures

**Figure 1:**
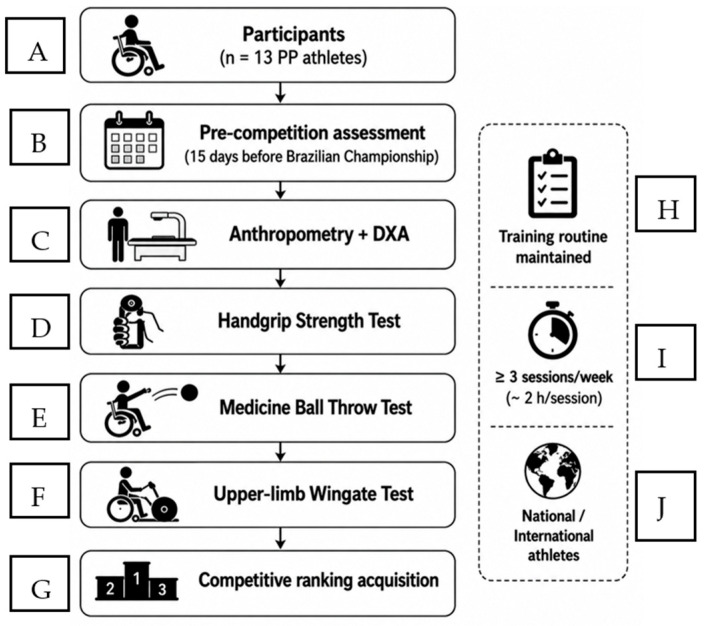
Study design and experimental procedures performed in Paralympic Powerlifting athletes, including participant characterization, pre-competition assessment period, sequence of experimental evaluations, and competitive ranking acquisition. A: Participants; B: Pre-competition assessment; C: Anthropometry + DXA; D: Handgrip Strength Test; E: Medicine Ball Throw Test; F: Upper-limb Wingate Test; G: Competitive ranking acquisition; H: Training routine maintained; I: A minimum training frequency of three sessions per week (~2 h per session); J: Participation in national and international competitions. Illustrations were generated/modified using AI-assisted tools exclusively for graphical representation purposes.

Assessments were conducted in an accessible, controlled laboratory setting with standardized equipment. Data collection occurred in a single session (~50 min per participant). Athletes were instructed to wear light clothing, empty their bladder prior to assessments, and avoid intense physical activity on the evaluation day (Figure 1).

The sequence of assessments was as follows:

1. Anthropometry (body mass and stature) and body composition analysis (DXA).
2. Upper-limb warm-up, followed by handgrip strength and medicine ball throw tests.
3. Ergometer familiarization and 5 min warm-up, followed by the Wingate upper-limb test.

All assessments were conducted by a qualified physical education team, following the specific protocols for each test.

#### 2.2.1. Anthropometry

Lean mass, fat-free mass, and fat mass were assessed using dual-energy X-ray absorptiometry (DXA; LUNAR^®^/GE PRODIGY—LNR 41.990, Washington, DC, USA; enCORE software, GE Healthcare^®^, version 15.0, Madison, WI, USA). DXA settings included: whole-body scan, voltage (kV): 76.0, current (mA): 0.150, and radiation dose (pGy): 0.4 (very low, no health risk).

Prior to DXA assessments, the equipment was calibrated daily according to the manufacturer’s recommendations. Participants were instructed to wear light clothing and avoid garments containing metallic components (e.g., zippers or metallic buttons). In addition, all metallic objects were removed before image acquisition to minimize potential interference during scanning procedures.

Assessments were performed with participants positioned in the supine position, with the arms extended alongside the body according to standardized procedures. Participants were instructed to empty their bladder before evaluation. However, fasting status and hydration control were not formally standardized before assessment. All evaluations were conducted by the same experienced examiner.

It should be noted that, in Paralympic populations, impairment-related characteristics such as asymmetries, amputations, contractures, and altered body positioning may influence body composition estimates derived from DXA assessments. Therefore, these factors should be considered when interpreting body composition findings in athletes with heterogeneous impairment profiles.

Body mass was measured using a digital scale (Filizola^®^, São Paulo, Brazil; max. 150 kg, accuracy 0.10 kg) or a wheelchair-accessible digital scale (Micheletti^®^, São Paulo, Brazil, max. 500 kg, min. 2 kg, accuracy 0.1 kg). Stature was measured using a stadiometer (Sanny^®^, São Paulo, Brazil; accuracy 0.1 mm) whenever orthostatic positioning was feasible. For athletes unable to maintain the orthostatic position due to impairment-related limitations, stature was estimated in the supine position (dorsal decubitus) according to standardized anthropometric procedures.

#### 2.2.2. Upper-Limb Isometric and Dynamic Strength

Isometric strength was assessed using a handgrip dynamometer (Jamar^®^—Sammons Preston Rolyan, Bolingbrook, IL, USA; scale: 0–90 kg, static indicator, five hand-size adjustments). Handgrip strength was selected due to its wide applicability in Paralympic populations, methodological feasibility, and use as an indicator of global isometric strength.

Assessments were performed with participants seated, with the arm positioned alongside the body and the elbow flexed at 90°. Familiarization procedures and individual grip adjustments were performed before testing. Three maximal trials were performed with each hand, with 60 s rest intervals between attempts. Both dominant and non-dominant hands were evaluated; however, the highest value obtained was recorded and expressed in kilogram-force (kgf) [25]. Consistent verbal encouragement was provided during all testing procedures.

Dynamic strength was assessed via a 2 kg medicine ball throw from a seated position with back support and stabilized feet. Each athlete performed three throws, and the longest distance (cm) was recorded using a tape measure from the front feet of the chair to the first ground contact point of the ball [26]. Participants were instructed to use only the arms and shoulder girdle. Upon signal, they explosively projected the ball as far as possible.

The medicine ball throw test was included as a complementary assessment of upper-limb dynamic performance due to its applicability in Paralympic populations, ease of implementation, and ability to provide information regarding explosive upper-limb actions. Although the movement pattern differs from Paralympic Powerlifting performance, the test was considered an indirect and complementary measure of upper-limb force production characteristics rather than a sport-specific assessment.

#### 2.2.3. Upper-Limb Anaerobic Power

Anaerobic power was evaluated using an arm-crank ergometer (Technogym S.p.A.^®^ Excite+ Top 700i, Gambettola, Italy) through the upper-limb Wingate test [26,27]. Unlike the traditional lower-limb Wingate protocol, in which resistance is fixed at 7.5% of body mass, the adapted protocol used the automatic resistance adjustment system provided by the ergometer software. Load determination was automatically individualized by the equipment according to participant characteristics entered into the system, specifically sex, age, and body mass, following the manufacturer’s integrated algorithm.

The protocol consisted of a 30 s all-out effort preceded by 5 min of warm-up and familiarization. Outcomes included peak power (W), mean power (W), relative peak power (W/kg), and relative mean power (W/kg). During the test, participants were instructed to maintain maximal effort throughout the 30 s protocol and received continuous verbal encouragement (e.g., motivational cues and time updates) to sustain performance. Cadence was visually monitored through the ergometer display during testing; however, cadence variables were not recorded because they were not considered study outcomes. All athletes were familiar with the testing procedure and successfully completed the protocol; therefore, no interruption criteria were required during assessments.

#### 2.2.4. Competitive Performance

Performance was determined by athletes’ official ranking positions at the national competition organized by the Brazilian Paralympic Committee [13]. Rankings were converted into an ordinal scale, with first place corresponding to the best performance. Additionally, we considered the Maximum Weight Lifted (Kg) during the competition as a measure of athletic performance.

### 2.3. Statistical Analysis

Data were analyzed using Jamovi^®^ software (version 2.3.18, Sydney, Australia). Descriptive statistics are presented as mean ± standard deviation (minimum; maximum).

Considering the relatively small sample size (*n* = 13), the heterogeneity of the participants, and the ordinal nature of the competitive ranking variable, associations between biodynamic variables and competitive performance indicators were examined using Spearman’s rank-order correlation coefficient (ρ). Correlation magnitudes were interpreted according to the following criteria [14]: trivial (<0.10), weak (0.10–0.39), moderate (0.40–0.69), strong (0.70–0.89), and very strong (≥0.90).

To provide an estimate of the precision of the observed associations, 95% confidence intervals (95% CI) were calculated for all correlation coefficients using Fisher’s z transformation and subsequently back-transformed to Spearman’s rho values. Confidence intervals were interpreted in conjunction with the correlation coefficients and adjusted *p*-values. Given the number of correlation analyses performed, the false discovery rate (FDR) was controlled using the Benjamini–Hochberg procedure. Accordingly, *p*-values obtained from the correlation analyses were adjusted to account for multiple comparisons, reducing the likelihood of Type I error while maintaining adequate statistical power for exploratory analyses.

Because maximum competition lift (kg) represents a continuous measure of lifting performance and reflects a distinct aspect of competitive performance from ranking position, additional exploratory simple linear regression analyses were performed as secondary analyses. This outcome was included to provide a direct quantification of the load successfully lifted during competition, complementing the ranking-based assessment of performance. Only variables that demonstrated significant associations with maximum competition lift in the exploratory Spearman correlation analyses after FDR correction were entered into the regression models.

To avoid overfitting due to the limited sample size, each independent variable was examined in an independent simple regression model, and no multivariable regression models were performed. For each regression model, the unstandardized regression coefficient (β), standard error (SE), 95% confidence interval (95% CI), coefficient of determination (R^2^), adjusted R^2^, and root mean square error (RMSE) were reported. Model assumptions were evaluated through analyses of residuals. Residual normality was assessed using the Shapiro–Wilk, Kolmogorov–Smirnov, and Anderson–Darling tests. Homoscedasticity was evaluated using the Breusch–Pagan, Goldfeld–Quandt, and Harrison–McCabe tests. Independence of residuals was examined using the Durbin–Watson statistic.

Statistical significance was established at *p* < 0.05 after Benjamini–Hochberg FDR correction for the correlation analyses and at *p* < 0.05 for the regression analyses.

## 3. Results

The characteristics of the sample are presented in Table 1, including age, duration of disability, type of disability, time in the sport, maximum lift in competition, total body mass, lean mass, body fat percentage, handgrip strength, medicine ball throw, peak and mean power, as well as their respective relative values.

Table 1 demonstrates substantial interindividual variability across anthropometric, strength, and anaerobic performance variables, reflecting the heterogeneous characteristics of Paralympic Powerlifting athletes included in the sample. Therefore, the observed correlations should be interpreted considering the potential influence of variability related to impairment profile, body mass, training background, and competitive level.

Based on Table 2, after applying the *p*-value correction, the relevant findings can be divided into two main groups. Regarding competition ranking, three significant inverse correlations were identified, indicating that higher values for these physical variables were associated with better sporting performance, reflected by a position closer to first place. Among these variables, Maximum Power showed a strong inverse correlation, Mean Power demonstrated a similar pattern, and Relative Mean Power exhibited the most pronounced association within this group, being classified as a very strong inverse correlation.

In contrast, when examining the outcomes related to the Maximum Competition Lift performance indicator (Table 2), two direct and statistically significant correlations were identified, suggesting that improvements in these physical capacities are associated with a greater ability to lift heavier loads during competition. Specifically, Total Lean Mass demonstrated a strong positive correlation with lifting performance, as did Left Handgrip Strength, which also showed a strong positive association.

Given the limited sample size and the heterogeneous characteristics of the participants, these associations should be interpreted with caution. The negative correlations indicate that higher power values were associated with better ranking positions (Table 2). Conversely, no statistically significant associations were observed between competitive performance and body composition variables, handgrip strength, or dynamic strength assessed by the medicine ball throw test. However, these findings should also be interpreted cautiously, considering the limited sample size, participant heterogeneity, and the potential influence of measurement specificity.

Based on the correlation analyses (Table 2), we selected the associations that remained significant after *p*-value correction and performed simple linear regression analyses using maximum competition lift (kg) as the performance indicator. This marker was chosen because it is a continuous scalar variable, unlike competition ranking, which represents an ordinal outcome.

**Figure 2:**
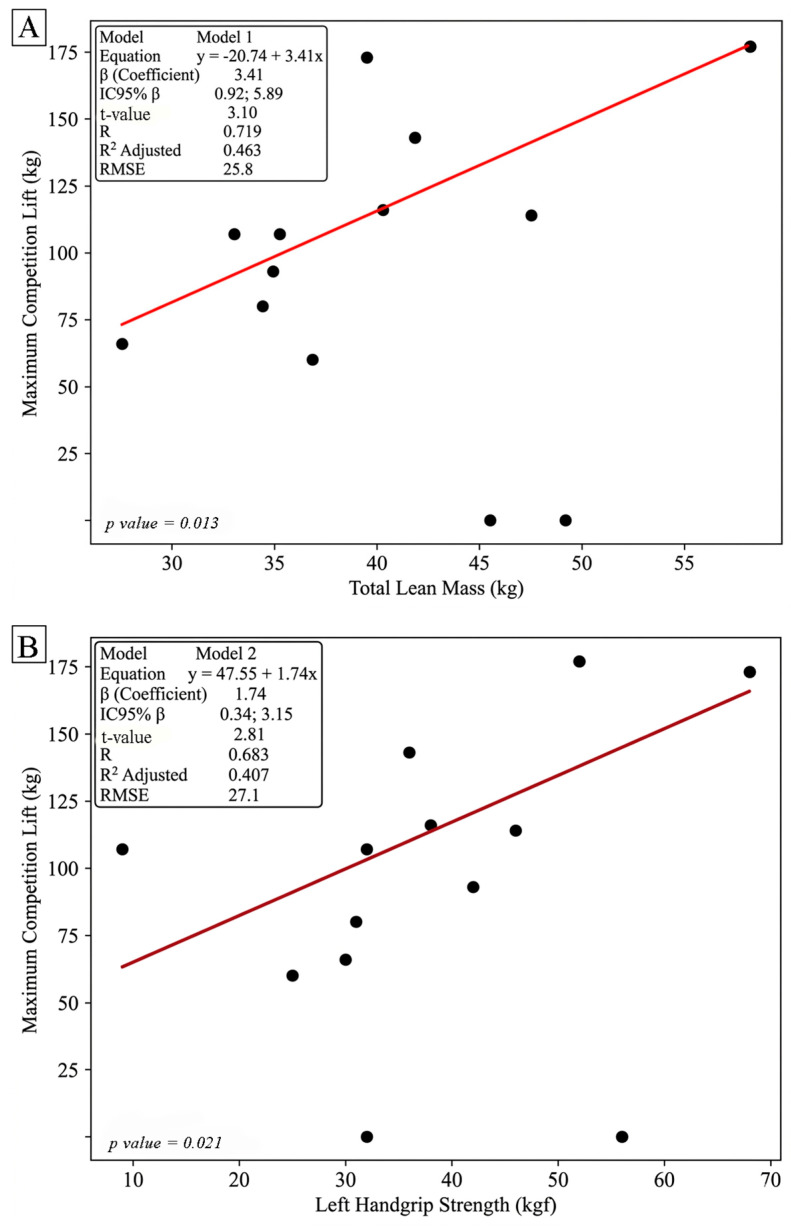
Simple linear regression analyses. (**A**) Model 1: Simple linear regression analysis between total lean mass and Maximum Competition Lift. (**B**) Model 2: Simple linear regression analysis between left-hand grip strength and Maximum Competition Lift. RMSE: root mean square error. IC 95%: 95% confidence interval.

As shown in Figure 2A, the β coefficient indicates that for every 1 kg increase in total lean mass, there is an absolute increase of 3.41 kg in maximum competition lift. The 95% confidence interval (95% CI: 0.92–5.89) suggests that, under the most conservative estimate, the increase is at least 0.92 kg, whereas under the most favorable estimate it may reach 5.89 kg for each additional kilogram of lean mass. Furthermore, total lean mass was associated with a substantial proportion of the variance observed in maximum competition lift within the present sample.

According to the β coefficient presented in Figure 2B, each additional 1 kg in left-hand grip strength was associated with an absolute increase of 1.74 kg in maximum competition lift. The 95% confidence interval indicates that this increase ranges from 0.34 to 3.15 kg (95% CI: 0.34–3.15). Similarly, left-hand grip strength was associated with a substantial proportion of the variability observed in maximum competition lift within the present sample. Given the exploratory nature of these analyses, the limited sample size (*n* = 13), and the heterogeneous characteristics of the participants, this regression estimates should be interpreted with caution.

## 4. Discussion

The present findings suggest that upper-limb anaerobic power may be associated with competitive performance in Paralympic Powerlifting athletes, partially supporting our initial hypothesis and indicating its potential relevance as a performance-related variable. Previous literature has consistently identified power as a key determinant of success in strength-dominant sports [3,6,9], and the current results extend this evidence to a Paralympic context. Although Paralympic Powerlifting is characterized by relatively controlled lifting velocities, the capacity to generate high levels of power appears to reflect underlying neuromuscular efficiency, rapid motor unit recruitment, and the ability to sustain high-intensity effort. Collectively, these factors may act as mediators between maximal strength expression and actual performance outcomes during competition. In addition, the results indicate that body composition and upper-limb isometric strength may also contribute to competitive performance, reinforcing the multifactorial nature of success in Paralympic Powerlifting.

### 4.1. Anaerobic Power and Competitive Performance in Paralympic Powerlifting

The analysis of power-related variables revealed that higher absolute peak power values (Table 2) were associated with superior competitive rankings, which may reflect the relevance of upper-limb power production during high-intensity lifting tasks in Paralympic Powerlifting [30]. However, these interpretations should be considered cautiously, since biomechanical variables such as bar velocity and specific lift-phase characteristics were not directly assessed in the present study. In parallel, relative peak power (Table 2) demonstrated strong correlations with ranking, reinforcing its potential value as a normalized performance-related indicator in weight-category sports [21]. This finding is particularly relevant in Paralympic contexts, where inter-individual variability in body mass and impairment characteristics may influence absolute outputs. However, competitive ranking in weight-category sports should be interpreted within the context of body-mass classification systems, since ranking outcomes may not exclusively reflect physiological differences between athletes.

Absolute mean power (Table 2) may reflect the ability to sustain power production throughout the exercise task, which could be relevant for performance during Paralympic Powerlifting competition [31]. Notably, relative mean power (Table 2) exhibited the strongest association with competition ranking, suggesting that the ability to maintain power output relative to body mass may be an important performance-related characteristic in this population. However, these findings should be interpreted with caution considering the correlational nature of the study, the limited sample size, and the heterogeneous characteristics of the participants. This observation is consistent with the notion that performance in weight-category sports may depend not only on absolute output but also on the efficiency with which force and power are produced relative to body mass.

### 4.2. Anaerobic Power and Sport Performance

The present findings are consistent with previous reports demonstrating associations between upper-limb anaerobic power—typically assessed via the Wingate test—and performance in Paralympic sports such as judo, swimming, and golf [18,19,20]. These observations may support the usefulness of the Wingate test as a functional assessment tool in sports involving short-duration, high-intensity upper-limb efforts [15,16,21]. However, comparisons across Paralympic sports should be interpreted with caution, as these modalities differ substantially in movement patterns, technical demands, competition structure, and physiological requirements. Furthermore, the transferability of Wingate-derived metrics to sport-specific performance may be influenced by differences in movement patterns, technical demands, and neuromuscular coordination. In addition, competitive performance in Paralympic Powerlifting depends on multiple factors that are not directly captured by the Wingate test, including technical proficiency, lifting strategy, competition experience, and sport-specific strength characteristics. Therefore, although Wingate-derived variables may provide useful information regarding anaerobic performance, they should be interpreted as complementary indicators rather than direct representations of competitive performance. To the best of our knowledge, this study is the first to directly examine the association between anaerobic power and competitive performance in Paralympic Powerlifting, thereby addressing a relevant gap in the literature.

### 4.3. Body Composition, Isometric and Dynamic Strength

Exploratory linear regression analyses indicated that lean mass was significantly associated with maximum competition lift (Figure 2A). This finding is consistent with previous studies conducted in able-bodied athletes, in which greater lean mass has been associated with superior performance in strength-related sports [10]. In the present study, each additional kilogram of lean mass was associated with an increase of 3.41 kg in maximum competition lift, and lean mass was associated with approximately 52% of the variance observed in maximum competition lift within the present sample. These findings suggest that lean tissue may contribute to force production capacity and competitive lifting performance, indicating that maintaining or increasing lean mass may be beneficial for Paralympic Powerlifting athletes.

In Paralympic athletes, the relevance of lean mass to performance may be attributed to its contribution to force production and torque generation during the execution of the competitive movement, despite the unique physiological and biomechanical characteristics associated with different impairment types [4,17,21,22,23]. Furthermore, the heterogeneity of impairments within the sample may have contributed to greater data variability, potentially reducing the sensitivity of correlation analyses while not preventing the identification of a significant association between lean mass and maximum competition lift. Collectively, these findings support the inclusion of body composition monitoring as part of long-term athlete development and performance management strategies in Paralympic Powerlifting.

Similarly, left-hand grip strength was significantly associated with maximum competition lift (Figure 2B). Although handgrip strength is commonly used as a surrogate marker of overall muscular strength [12], our findings suggest that this measure may provide relevant information regarding functional capacity associated with performance in Paralympic Powerlifting. Specifically, each additional kilogram-force of handgrip strength was associated with an increase of 1.74 kg in maximum competition lift and was associated with approximately 47% of the variance observed in maximum competition lift within the present sample.

The observed association between grip strength and maximum competition lift may reflect the importance of upper-limb stability, neuromuscular control, and overall force-generating capacity required for the successful execution of the competitive bench press. Although handgrip strength does not directly replicate the competitive movement, it may represent a practical and low-cost indicator of neuromuscular function that can be easily implemented in routine athlete monitoring. In contrast, medicine ball throw performance was not significantly associated with competitive performance, further emphasizing the importance of biomechanical specificity in performance assessment, as this test may not adequately reproduce the force–time characteristics and technical demands of the bench press movement.

The apparent discrepancy between the correlation and regression findings may be explained by the distinct nature of the performance outcomes examined. Maximum competition lift represents a direct measure of lifting capacity, whereas ranking position is influenced by additional contextual factors, including body-weight category, competitive field, and competition-specific dynamics. Consequently, physiological variables associated with lifting performance may not necessarily translate into superior ranking outcomes. Because these regression analyses were exploratory and conducted in a small and heterogeneous sample, the observed associations should be interpreted cautiously and confirmed in future studies with larger and more homogeneous cohorts.

### 4.4. Limitations and Future Directions

Some aspects should be considered when interpreting the present findings. The study was conducted with a relatively small sample of Paralympic Powerlifting athletes presenting different impairment characteristics and competitive backgrounds, which reflects the nature of the population investigated. Additionally, competitive performance was assessed through ranking position, a measure that may be influenced by factors beyond physiological characteristics alone, particularly in weight-category sports. Although significant associations with maximum competition lift were identified, the limited sample size precluded the use of multivariable regression models capable of adjusting for potential confounding factors such as sex, body-weight category, impairment type, and training experience. Therefore, the observed associations should be interpreted within the context of the study design and participant characteristics.

Despite these considerations, the findings provide relevant information regarding the association between anaerobic power and competitive performance in Paralympic Powerlifting athletes, while also highlighting the potential contribution of lean mass and upper-limb isometric strength to lifting performance. Future research should adopt longitudinal and intervention-based designs to better elucidate causal mechanisms and training adaptations. The integration of biomechanical analyses (e.g., bar velocity profiling), neuromuscular assessments (e.g., electromyography), and advanced modeling approaches may provide a more comprehensive understanding of performance determinants. Furthermore, exploring the interaction among anaerobic power, body composition, strength characteristics, and technical execution could offer valuable insights for optimizing training strategies in this population.

### 4.5. Practical Applications

From an applied perspective, the present findings suggest that anaerobic power, lean mass, and upper-limb isometric strength represent relevant performance-related variables in Paralympic Powerlifting. The use of validated assessment tools, such as the upper-limb Wingate test and handgrip dynamometry, enables practitioners to systematically evaluate athletes’ readiness and monitor physiological adaptations over time. Importantly, training interventions aimed at maintaining lean mass and enhancing the rate of force development, overall muscular strength, and power output—while preserving technical proficiency—may contribute to improved competitive outcomes. These findings may also support evidence-based approaches to talent identification, athlete monitoring, and long-term athlete development within Paralympic sport systems.

## 5. Conclusions

In conclusion, upper-limb anaerobic power assessed through the Wingate test showed strong associations with competitive performance in Paralympic Powerlifting athletes, suggesting its potential relevance as a performance-related variable in the sport. Notably, relative mean power exhibited the strongest association with competitive ranking, suggesting that higher relative power outputs may be associated with superior competitive placements. These findings reinforce the importance of considering normalized performance metrics in weight-category sports, where performance efficiency may be more informative than absolute outputs alone.

In addition, exploratory linear regression analyses indicated significant associations between total lean mass, left-hand grip strength, and maximum competition lift. These findings suggest that body composition and upper-limb isometric strength may contribute to competitive performance through mechanisms related to force production capacity and neuromuscular function. Collectively, these findings suggest that competitive performance in Paralympic Powerlifting may be associated with multiple physiological characteristics, including anaerobic power, muscular strength, and body composition, rather than any single factor in isolation.

From a practical perspective, the present findings may support the inclusion of anaerobic power assessments, body composition monitoring, and handgrip strength evaluation as part of routine athlete assessment and monitoring programs in Paralympic Powerlifting. Training strategies aimed at optimizing lean mass, muscular strength, and power output—while maintaining technical proficiency—may contribute to improved competitive performance.

However, given the exploratory nature of the study, the limited sample size, participant heterogeneity, and cross-sectional design, these findings should be considered preliminary and interpreted with caution. Future studies involving larger and more homogeneous cohorts of Paralympic Powerlifting athletes are needed to confirm these associations and further clarify the factors associated with competitive performance.

## Figures and Tables

**Table 1:** Sample characterization.

Variable	Total Sample (*n* = 13)	
	Average ± SD	Min; Max
Age (years)	34.4 ± 10.6	19.0; 55.0
Time with disability (years)	26.6 ± 10.8	11.0; 50.0
Time in the sport (years)	9.3 ± 8.4	2.0; 27.0
Maximum competition lift (Kg)	112.3 ± 38.9	60.0; 177.0
Total mass (kg)	67.6 ± 18.6	45.0; 105.9
Lean mass (Kg)	40.3 ± 8.1	27.5; 58.2
% FAT	36.7 ± 9.6	19.7; 54.1
Right handgrip strength (Kgf)	42.2 ± 15.0	14.0; 74.0
Left handgrip strength (Kgf)	38.2 ± 14.9	9.0; 68.0
Maximum throw (cm)	480.3 ± 153.5	230.0; 840.0
Maximum power (W)	227.2 ± 136.6	20.0; 441.0
Relative maximum power (W/kg)	3.5 ± 2.1	0.2; 7.4
Average power (W)	185.7 ± 114.9	20.0; 380.0
Relative average power (W/kg)	2.9 ± 1.7	0.2; 6.4
Type of disability	n	%
Congenital	04	30.7
Acquired	09	69.3

Kg—kilogram, n—number, %—percentage, cm—centimeter, W—Watts, W/Kg—Watts per kilogram. Kgf: kilogram-force.

**Table 2:** Spearman correlations between biodynamic variables and sports performance indicators (Competition Ranking and Maximum Competition Lift [kg]).

Variable	Ranking				Maximum Competition Lift (Kg)			
	ρ	ρ CI 95%	*p*	*p* Adjusted	ρ	ρ CI 95%	*p*	*p* Adjusted
Maximum Competition Lift (Kg)	−0.461	−0.807; 0.121	0.154	0.294	—	—	—	—
Maximum Power (W)	**−0.738**	**−0.916; 0.315**	0.009	**0.0378**	0.623	0.110; 0.874	0.040	0.0933
Relative Maximum Power (W/kg)	−0.693	−0.900; −0.230	0.018	0.0570	0.260	−0.340; 0.709	0.441	0.5448
Mean Power (W)	**−0.794**	**−0.936; −0.432**	**0.004**	**0.0378**	0.687	0.219; 0.898	0.019	0.0570
Relative Mean Power (W/kg)	**−0.859**	**−0.957; −0.585**	**<0.001**	**0.0210**	0.337	−0.263; 0.749	0.311	0.4354
Fatigue Index (%)	−0.298	−0.729; 0.303	0.373	0.4896	0.211	−0.385; 0.683	0.532	0.6058
Total Body Mass (kg)	−0.097	−0.615; 0.480	0.777	0.7770	0.387	−0.208; 0.773	0.239	0.3585
Total Lean Mass (Kg)	−0.189	−0.670; 0.404	0.577	0.6058	**0.743**	**0.325; 0.918**	**0.009**	**0.0378**
Right Handgrip Strength (Kgf)	−0.393	−0.776; 0.202	0.232	0.3585	0.661	0.173; 0.888	0.027	0.0709
Left Handgrip Strength	−0.397	−0.778; 0.197	0.226	0.3585	**0.747**	**0.333; 0.920**	**0.008**	**0.0378**
Medicine Ball Throw Distance (cm)	−0.201	−0.677; 0.394	0.553	0.6058	0.553	0.003; 0.846	0.078	0.1638

**Bold**: Statistically Significant *p* < 0.05. (Kg): Kilogram. (W): Watts. (W/Kg): Relative watts. (%): Percent. (Kgf): Kilogram-force. (cm): Centimeter. ρ: Spearman’s rho. 95% CI: 95% confidence interval.

## Data Availability

The database for this study is publicly available at: https://figshare.com, under the Doi: https://doi.org/10.6084/m9.figshare.32652762.

## References

[1] International Paralympic Committee World Para Powerlifting Rules and Regulations. https://www.paralympic.org/powerlifting/rules.

[2] Bompa T.O., Buzzichelli C.A. (2019). Periodização: Teoria e Metodologia do Treinamento.

[3] Oliveira J.I.V., de Lucena E.G.P., Ferland P.-M., Oliveira S.F.M., Uchida M.C. (2024). Para Powerlifting Performance: A Systematic Review. Int. J. Sports Med..

[4] Teles L.J.L., Aidar F.J., Matos D.G.d., Marçal A.C., Almeida-Neto P.F.d., Neves E.B., Moreira O.C., Ribeiro Neto F., Garrido N.D., Vilaça-Alves J. (2021). Static and Dynamic Strength Indicators in Paralympic Power-Lifters with and without Spinal Cord Injury. Int. J. Environ. Res. Public Health.

[5] Aidar F.J., Cataldi S., Badicu G., Silva A.F., Clemente F.M., Latino F., Greco G., Fischetti F. (2022). Paralympic Powerlifting as a Sustainable Way to Improve Strength in Athletes with Spinal Cord Injury and Other Disabilities. Sustainability.

[6] Aidar F.J., Brito C.J., de Matos D.G., de Oliveira L.A.S., de Souza R.F., de Almeida-Neto P.F., de Araújo Tinoco Cabral B.G., Neiva H.P., Neto F.R., Reis V.M. (2022). Force–velocity relationship in Paralympic Powerlifting: Two or multiple-point methods to determine a maximum repetition. BMC Sports Sci. Med. Rehabil..

[7] Antolinez A.K., Edwards P.F., Holmes M.W.R., Beaudette S.M., Button D.C. (2024). The Effects of Load, Crank Position, and Sex on the Biomechanics and Performance during an Upper Body Wingate Anaerobic Test. Med. Sci. Sports Exerc..

[8] Nowak A.M., Molik B. (2021). Application of the Arm-Cranking 30-Second Wingate Anaerobic Test (WAnT) to Assess Power in Amputee Football Players. Acta Bioeng. Biomech..

[9] Lovell D., Mason D., Delphinus E., Eagles A., Shewring S., McLellan C. (2011). Does Upper Body Strength and Power Influence Upper Body Wingate Performance in Men and Women?. Int. J. Sports Med..

[10] Cherif M., Said M., Bannour K., Alhumaid M.M., Ben Chaifa M., Khammassi M., Aouidet A. (2022). Anthropometry, Body Composition, and Athletic Performance in Specific Field Tests in Paralympic Athletes with Different Disabilities. Heliyon.

[11] O’Connor S.R., Fagher K., Williamson S., Pluim B.M., Ardern C.L., Janse van Rensburg D.C., Heron N. (2022). Assessment of Muscle Strength in Para-Athletes: A Systematic Review of Observational Studies. Sports Med. Health Sci..

[12] Vaishya R., Misra A., Vaish A., Ursino N., D’Ambrosi R. (2024). Hand Grip Strength as a Proposed New Vital Sign of Health: A Narrative Review of Evidence. J. Health Popul. Nutr..

[13] Comitê Paralímpico Brasileiro Resultados do Campeonato Brasileiro de Halterofilismo—2024. https://cpb.org.br/competicoes/campeonato-brasileiro/brasileiros-2024/.

[14] Schober P., Boer C., Schwarte L.A. (2018). Correlation Coefficients: Appropriate Use and Interpretation. Anesth. Analg..

[15] Lovell D., Farley A., Wiegand A.N., Solomon C., Harvey L., McLellan C. (2013). The Contribution of Energy Systems during the Upper Body Wingate Anaerobic Test. Appl. Physiol. Nutr. Metab..

[16] Puce L., Trabelsi K., Trompetto C., Ramadan J., Ammar A., Drid P., Paoli A., Chtourou H., Chaabene H. (2022). A Bibliometrics-Enhanced, PAGER-Compliant Scoping Review of the Literature on Paralympic Powerlifting: Insights for Practices and Future Research. Healthcare.

[17] Gee C.M., Lacroix M.A., Stellingwerff T., Gavel E.H., Logan-Sprenger H.M., West C.R. (2021). Physiological Considerations to Support Podium Performance in Para-Athletes. Front. Rehabil. Sci..

[18] Šimenko J., Mahnič N., Kukovica D., Sertić H., Segedi I., Milić R., Karpljuk D., Ceylan B., Rauter S. (2024). Exploring the Relationship between Anaerobic and Morphological Characteristics and Competition Success in Young Male Slovenian Judo Athletes. Appl. Sci..

[19] Sorbie G.G., Glen J., Richardson A.K. (2021). Positive Relationships Between Golf Performance Variables and Upper Body Power Capabilities. J. Strength Cond. Res..

[20] Cavedon V., Rosponi A., Alviti F., De Angelis M., Guerra E., Rodio A., Di Giacinto B., Milanese C., Bernardi M. (2021). Comparison between the 10- and the 30-s-Long Wingate Anaerobic Test in Summer Paralympic Athletes with a Lower Limb Impairment. Sport Sci. Health.

[21] Buhmann R., Sayers M., O’Brien J., Borg D. (2024). Important Features of Bench Press Performance in Non-Disabled and Para Athletes: A Scoping Review. PLoS ONE.

[22] Severin C., Baumgart J.K., Haugen T., Hogarth L. (2023). Peak Age and Performance Trajectories in Para Powerlifters. Am. J. Phys. Med. Rehabil..

[23] Neto R.F., Dorneles J.R., Luna R.M., Ferreira-Júnior J.B., da Silva-Grigoletto M.E., Aidar F.J., dos Santos J.L., de Oliveira V.M.A., Almeida-Neto P.F. (2022). Performance Differences Between the Arched and Flat Bench Press in Beginner and Experienced Paralympic Powerlifters. J. Strength Cond. Res..

[24] Faul F., Erdfelder E., Lang A.-G., Buchner A. (2007). G*Power 3: A Flexible Statistical Power Analysis Program for the Social, Behavioral, and Biomedical Sciences. Behav. Res. Methods.

[25] Gorla J.I., Campana M.B., Oliveira L.Z. (2009). Teste e Avaliação em Esporte Adaptado.

[26] Gorla J.I. (2020). Manual de Medidas e Avaliação Físico-Motora: Deficiência Física.

[27] Bar-Or O. (1987). The Wingate Anaerobic Test: An Update on Methodology, Reliability and Validity. Sports Med..

[28] Kraemer W.J., Ratamess N.A. (2004). Fundamentals of Resistance Training: Progression and Exercise Prescription. Med. Sci. Sports Exerc..

[29] McKay A.K.A., Stellingwerff T., Smith E.S., Martin D.T., Mujika I., Goosey-Tolfrey V.L., Sheppard J., Burke L.M. (2022). Defining Training and Performance Caliber: A Participant Classification Framework. Int. J. Sports Physiol. Perform..

[30] Aidar F.J., Clemente F.M., Matos D.G., Marçal A.C., de Souza R.F., Moreira O.C., Almeida-Neto P.F., Vilaça-Alves J., Garrido N.D., dos Santos J.L. (2021). Evaluation of Strength and Muscle Activation Indicators in Sticking Point Region of National-Level Paralympic Powerlifting Athletes. J. Funct. Morphol. Kinesiol..

[31] Santos L.C.V., Aidar F.J., Villar R., Greco G., de Santana J.L., Marçal A.C., de Almeida-Neto P.F., de Araújo Tinoco Cabral B.G., Badicu G., Nobari H. (2023). Evaluation of the Training Session in Elite Paralympic Powerlifting Athletes Based on Biomechanical and Thermal Indicators. Sports.

